# Rare variants in *NRSN2* cause non-syndromic orofacial cleft through dysregulation of TGF-β signaling

**DOI:** 10.1016/j.gendis.2025.101865

**Published:** 2025-09-23

**Authors:** Xiaowen Zheng, Xuqin Liang, Xiantao Wu, Qing He, Chunyan Yin, Yuhua Jiao, Yanhao Wang, Yuxia Hou, Yi Ding, Huaxiang Zhao

**Affiliations:** a Department of Orthodontics, Shanghai Ninth People’s Hospital, College of Stomatology, Shanghai Jiao Tong University School of Medicine National Clinical Research Center for Oral Disease Shanghai Key Laboratory of Stomatology & Shanghai Research Institute of Stomatology, Shanghai 200011, China; b Key Laboratory of Shaanxi Province for Craniofacial Precision Medicine Research, College of Stomatology, Xi'an Jiaotong University, Xi'an, Shaanxi 710004, China; c Department of Pediatrics, The Second Affiliated Hospital of Xi'an Jiaotong University, Xi'an, Shaanxi 710004, China; d Department of Physiology and Pathophysiology, School of Basic Medical Sciences, Xi'an Jiaotong University, Xi'an, Shaanxi 710061, China; e Department of Orthodontics, College of Stomatology, Xi'an Jiaotong University, Xi'an, Shaanxi 710004, China

Orofacial cleft (OFC) is the most common congenital craniofacial disorder that significantly affects the appearance and orofacial function of patients. Although previous studies have demonstrated that rare variants are significant contributors to the genetic etiology of non-syndromic OFC (NSOFC),^1^ only in a limited number of cases the underlying causal genes are identified. The human Neurensin 2 (*NRSN2*) gene, encoding a two-transmembrane-domain protein,^2^ has not previously been associated with craniofacial development or malformations. In this study, we performed whole-exome sequencing (WES) and target-region sequencing (TRS) on 10 multiplex families and 138 sporadic cases with NSOFC, identifying three rare variants in the *NRSN2* gene. Functional analyses in mammalian cells revealed that NRSN2 interacts with and degrades type 1 and type 2 TGF-β receptors (TβRI and TβRII) through the endosome–lysosome pathway, thereby inhibiting TGF-β signaling. In contrast, all three *NRSN2* variants show an impaired capacity to degrade TβRI and TβRII, causing unrestrained TGF-β signaling. Consistently, these *NRSN2* variants are less effective to induce craniofacial abnormalities in zebrafish embryos, corroborating that they are loss-of-function (LoF) mutations. Taken together, we conclude that *NRSN2* variants behave as hypomorphic alleles that result in aberrant TGF-β signaling and thus *NRSN2* is a novel causal gene for NSOFC in humans.

We first recruited 10 multiplex families with NSOFC and performed WES to uncover potential causal genes/variants in these families. In one of the families, we narrowed down to 16 most likely candidate genes/variants using filtering criteria as described previously^3^ (Fig. S1 and Table S1). Among these remaining variants, we observed a heterozygous frameshift variant in the *NRSN2* gene (c.171_172delGC/p.W57CfsX38, referred to as p.W57fs), which has not previously been associated with craniofacial development or abnormalities. Both patients in this multiplex family exhibited a cleft lip, but no cleft palate or other significant malformations were detected (Fig. 1A and Table S2). Next, to validate the association between rare *NRSN2* variants and NSOFC, we performed TRS on 138 sporadic NSOFC cases, and two additional rare heterozygous missense variants in the *NRSN2* gene were identified: c.311G > A/p.R104Q and c.549C > G/p.F183L (referred to as p.R104Q and p.F183L, respectively). Notably, both sporadic cases also exhibited cleft lips, similar to the patients in Family 1 (Fig. 1B and Table S2). All three variants identified through next-generation sequencing were further confirmed via PCR-Sanger sequencing (Fig. S2).

**Figure 1:**
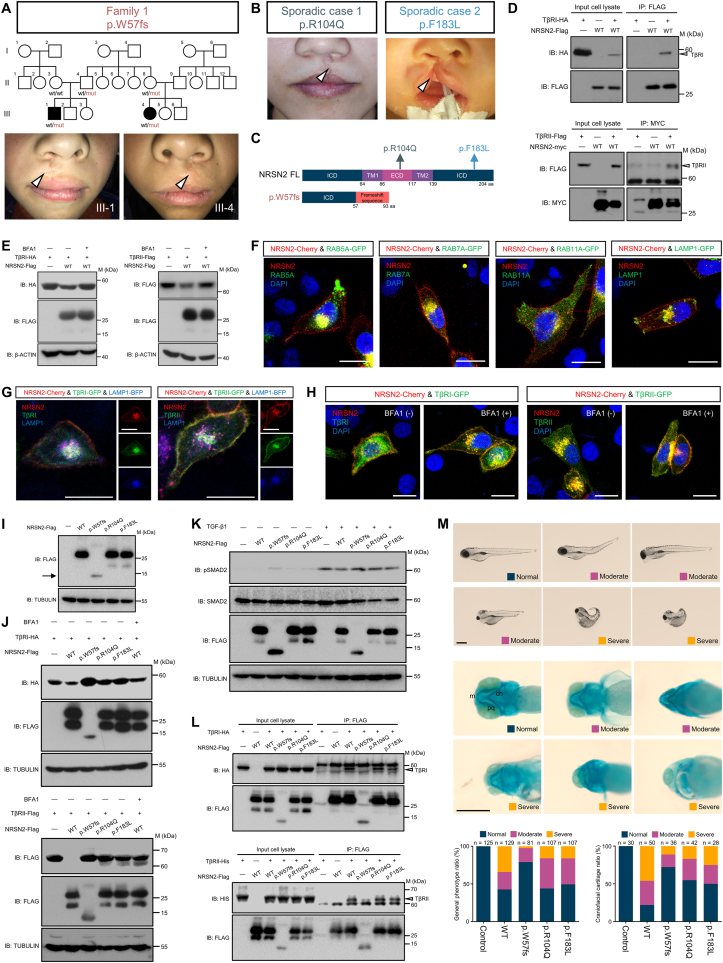
Rare variants in *NRSN2* cause non-syndromic orofacial cleft through dysregulation of TGF-β signaling. **(A)** The pedigree map and clinical photos of the multiplex family in which the p.W57fs variant has been identified. Circles indicate female members while squares male members; blank symbols indicate unaffected members while black symbols patients with NSOFC. White arrowheads indicate regions affected by clefts. **(B)** Clinical photos of two sporadic NSOFC cases, where the p.R104Q and p.F183L variants have been identified. White arrowheads indicate cleft-affected regions. **(C)** Location of NRSN2 variants in the protein. FL, full-length; ICD, intracellular domain; ECD, extracellular domain; TM, transmembrane domain. **(D)** NRSN2 can interact with TβRI and TβRII. HEK-293T cells were transfected with the indicated constructs. Cells were harvested for immunoprecipitation (IP) with anti-FLAG (upper panel) and anti-MYC (lower panel) antibodies, followed by immunoblotting (IB). **(E)** NRSN2 reduced the expression of TβRI (left panel) and TβRII (right panel), while the lysosome inhibition rescued their expression. HEK-293T cells were transfected with the indicated constructs. Four hours before being harvested, the cells were treated with or without the lysosome inhibitor bafilomycin A1 (BFA1, 1 μM; MCE, #HY-100558). **(F)** NRSN2 showed strong co-localization with early and late endosomes as well as lysosomes, but weaker co-localization with recycling endosomes. HeLa cells were transfected with the indicated constructs. Yellow indicates co-localization of NRSN2 with RAB5A/RAB7A/RAB11A/LAMP1. RAB5A, early endosome marker; RAB7A, late endosome marker; RAB11A, recycling endosome marker; LAMP1, lysosome marker. Scale bars, 20 μm. **(G)** Strong co-localization signals were observed among NRSN2, LAMP1, and TβRI/TβRII. HeLa cells were transfected with the indicated constructs. White indicates co-localization of NRSN2, LAMP1, and TβRI/TβRII. Scale bars, 20 μm. **(H)** BFA1-mediated lysosomal inhibition enhanced the co-localization of NRSN2 with TβRI/TβRII on the plasma membrane. HeLa cells were transfected with the indicated constructs and treated with or without 1 μM BFA1 for 4 h before being harvested. Yellow indicates co-localization of NRSN2 and TβRI/TβRII. Scale bars, 20 μm. **(I)** The p.W57fs mutant exhibited lower expression compared to WT NRSN2, while p.R104Q and p.F183L showed similar expression levels to WT. HeLa cells were transfected with the indicated constructs. The arrow indicates p.W57fs NRSN2. **(J)** All three variants failed to degrade TβRI (upper panel) and TβRII (lower panel) compared to the WT NRSN2. HEK-293T cells were transfected with the indicated constructs, treated with or without 1 μM BFA1 for 4 h before being harvested. **(K)** WT NRSN2 attenuated TGF-β1-induced phospho-SMAD2 (pSMAD2), while the p.W57fs, p.R104Q, and p.F183L variants impaired this inhibitory function. HeLa cells transfected with the indicated constructs were treated with or without 100 pM TGF-β1 for 30 min. **(L)** The p.W57fs variant showed a significant reduction in interaction with TGF-β receptors, whereas p.R104Q and p.F183L had no significant effect on the interaction of NRSN2 with TβRI and TβRII. HEK-293T cells were transfected with the indicated constructs and harvested for IP-IB. **(M)** p.W57fs, p.R104Q, and p.F183L mutant NRSN2 were less active to induce craniofacial abnormalities in zebrafish embryos. The general phenotypes (upper panel) and craniofacial cartilage phenotypes (middle panel) of zebrafish embryos at 5 days post-fertilization (dpf) after microinjection of 400 pg mRNA of WT, p.W57fs, p.R104Q or p.F183L *NRSN2* at the one-cell stage are shown. The phenotypes were classified as “normal”, “moderate”, and “severe”. Lower panel, quantification of embryonic phenotypes. The number of embryos analyzed for each condition is indicated on the top of each column. Scale bar, 500 μm.

Among the three variants, p.R104Q and p.F183L are reported as low frequency in various databases, while p.W57fs appears to be novel as it has not been documented in any databases (Table S2). We conducted PCR-Sanger sequencing of the p.W57fs variant in 102 unaffected Chinese individuals, and none in this control cohort carried the variant, thereby validating its rarity (Supplemental Material 1). The mutant sites of p.R104Q and p.F183L are situated at the extracellular and intracellular regions of NRSN2, respectively, while p.W57fs not only leads to truncation of the NRSN2 protein but also introduces a frameshift (Fig. 1C).

Given that NRSN2 is a transmembrane protein, we used AlphaFold-3 to model the complex structure between NRSN2 and the transmembrane receptor proteins involved in signaling pathways critical to OFC occurrence.^4^ Our analysis showed that the transmembrane (TM) domain of NRSN2 forms a stable complex with the TM domains of TβRI and TβRII (Fig. S3), key components of TGF-β signaling implicated in OFC,^5^ and co-immunoprecipitation (co-IP) experiments confirmed their interaction with wild-type NRSN2 (Fig. 1D).

During the co-IP assays described above, we observed a reduction in TβRI/TβRII expression when co-transfected with NRSN2 (Fig. 1D), leading us to hypothesize that NRSN2 might degrade TβRI and TβRII. To test this, we performed receptor degradation experiments. HEK-293T cells were co-transfected with NRSN2 and TβRI/TβRII, and the immunoblotting (IB) results showed that NRSN2 reduced the expression of both TβRI and TβRII (Fig. 1E), indicating that NRSN2 facilitates the degradation of these receptors.

Since previous studies suggested that NRSN2 may localize to lysosomes,^2^ we further investigated whether NRSN2 degrades TβRI/TβRII via the endosome–lysosome pathway. Immunofluorescence (IF) was performed to analyze the co-localization of NRSN2 with several endosomal and lysosomal markers. NRSN2 showed strong co-localization with early (RAB5A) and late (RAB7A) endosomal markers as well as a lysosomal marker (LAMP1), but weaker co-localization with a recycling (RAB11A) endosomal marker (Fig. 1F). Furthermore, co-localization assays involving NRSN2, LAMP1, and TβRI/TβRII revealed strong co-localization signals among all three proteins (Fig. 1G). Additionally, we observed that lysosomal inhibition with bafilomycin A1 (BFA1) rescued the expression of TβRI and TβRII, which was degraded by NRSN2 (Fig. 1E). IF assays corroborated these results, showing enhanced co-localization of TβRI/TβRII with NRSN2 on the plasma membrane in BFA1-treated cells compared to those in untreated cells (Fig. 1H).

To examine the pathogenicity of these variants, we conducted functional experiments. The p.W57fs variant exhibited a lower expression level compared to the wild-type NRSN2, while p.R104Q and p.F183L did not affect NRSN2 expression (Fig. 1I). In receptor degradation assays, all three variants, unlike wild-type NRSN2, failed to degrade TβRI and TβRII (Fig. 1J), indicating that they were LoF variants. Overexpression of wild-type NRSN2 attenuated the TGF-β1-induced phosphorylation of SMAD2 (pSMAD2) in HeLa cells, verifying NRSN2 as an inhibitory regulator of TGF-β signaling. In contrast, the p.W57fs, p.R104Q, and p.F183L variants impaired this inhibitory function of NRSN2, further confirming their LoF nature (Fig. 1K).

We then explored whether these variants affect the interactions between NRSN2 and TβRI/TβRII. Co-IP experiments in HEK-293T cells revealed a significant reduction in the interaction between the p.W57fs variant and TGF-β receptors, consistent with the lower expression of the p.W57fs variant. However, neither the p.R104Q nor the p.F183L variant had a significant effect on NRSN2 binding to TβRI/TβRII (Fig. 1L), and the biochemical mechanisms by which these missense variants influence NRSN2 function require further investigation.

We microinjected mRNAs encoding human wild-type *NRSN2* or its variants into one-cell stage zebrafish embryos. Injection of wild-type *NRSN2* mRNA resulted in varying degrees of developmental abnormalities. In comparison, the p.W57fs, p.R104Q, and p.F183L variants showed a reduced capacity to induce developmental abnormalities. To further corroborate our findings, we utilized Alcian blue staining to visualize the craniofacial cartilage. Consistently, less than 1/4 of the embryos injected with wild-type *NRSN2* mRNA exhibited normal craniofacial cartilage, whereas the p.W57fs, p.R104Q, and p.F183L variants induced fewer craniofacial defects, with p.W57fs exhibiting the least potency (Fig. 1M). We further performed whole-mount *in situ* hybridization and IF for Col2a1a, a chondrocyte marker, to validate the differential effects of wild-type and mutant *NRSN2*, and found that all three variants showed a reduced ability to repress Col2a1a expression (Fig. S4).

Collectively, these functional experiments demonstrate that p.W57fs, p.R104Q, and p.F183L are LoF variants that impair the function of NRSN2 both *in vitro* and *in vivo*; therefore, all three *NRSN2* variants are classified as pathogenic (Table S3) according to the ACMG guidelines. However, a limitation of this study is the relatively small sample size, and further research involving larger cohorts is needed.

In summary, we propose a model elucidating how NRSN2 influences craniofacial development and the formation of OFC (Fig. S5). Under normal physiological conditions, wild-type NRSN2 interacts with TβRI and TβRII, facilitating their degradation through the endosome–lysosome pathway. This regulatory mechanism maintains the appropriate intensity of TGF-β signaling (left part in Fig. S5). However, the identified variants exhibit impaired function compared to wild-type NRSN2, resulting in aberrantly enhanced TGF-β signaling, which ultimately contributes to the formation of OFC (right part in Fig. S5).

## CRediT authorship contribution statement

**Xiaowen Zheng:** Writing – review & editing, Writing – original draft, Resources, Methodology, Investigation, Funding acquisition. **Xuqin Liang:** Writing – review & editing, Writing – original draft, Methodology, Investigation. **Xiantao Wu:** Methodology, Investigation. **Qing He:** Methodology, Investigation. **Chunyan Yin:** Investigation, Writing – review & editing. **Yuhua Jiao:** Methodology, Investigation. **Yanhao Wang:** Methodology, Investigation. **Yuxia Hou:** Writing – original draft, Resources, Funding acquisition, Conceptualization. **Yi Ding:** Writing – review & editing, Writing – original draft, Supervision, Resources, Conceptualization. **Huaxiang Zhao:** Writing – review & editing, Writing – original draft, Supervision, Resources, Funding acquisition, Conceptualization.

## Ethics declaration

This work was approved by the Ethics Committee of Hospital of Stomatology, Xi'an Jiaotong University (xjkqll[2019]NO.014), and informed consent was obtained from the participants or their guardians. All zebrafish experiments were approved by the Committee on the Ethics of Animal Experiments of Xi'an Jiaotong University (No. XJTULAC2020-411).

## Funding

This work was supported by the 10.13039/501100001809National Natural Science Foundation of China (No. 82370909, 82001030, 81901029, 82572120), Shanghai Sailing Program (China) (No. 19YF1426400), Key Research and Development Project of the Shaanxi Province Health Scientific Research and Innovation Capacity Enhancement Program (China) (No. 2025YF-05), and Shaanxi Province Youth Science and Technology Star Program (China) (No. 2025ZC-KJXX-140).

## Conflict of interests

The authors declare no conflict of interests.
