# Supplementary material for: Rare variants in NRSN2 cause non-syndromic orofacial cleft through dysregulation of TGF-β signaling
Source: Genes Dis. 2025 Sep 23;13(3):101865. doi: 10.1016/j.gendis.2025.101865 (PMC12854861; doi:10.1016/j.gendis.2025.101865)
Supplement: Multimedia component 1 [file mmc1.pdf]

**<u>Supplemental Material 1</u>**

# Sanger sequencing of *NRSN2* p.W57fs variant in 102 unaffected Han Chinese people

## Summary


- Information of NRSN2 p.W57fs variant: chr20:330457GGC>G/c.171_172delGC/p.W57fs.
- PCR primers: F-GCTATGCGTGTAGGGTG, R-GGGTTTTCTGTTGCTTG, product size-462 bp.
- 102 samples were sequenced, and all samples were wild-type.


## Sanger sequencing results

| Sample-1 | Sample-2 | Sample-3 |
|---|---|---|
| 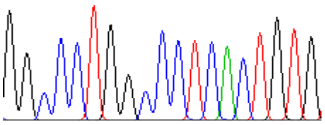 | 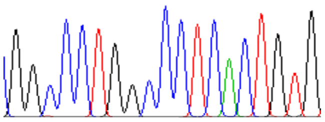 | 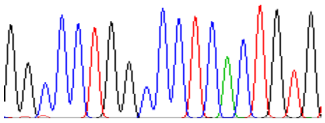 |
| Sample-4 | Sample-5 | Sample-6 |
| 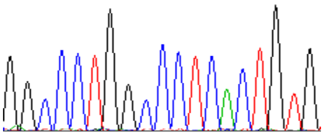 | 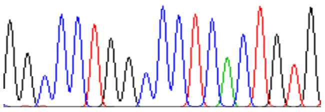 | 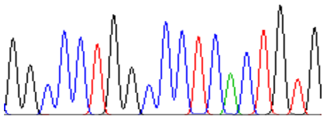 |

| Sample-7 | Sample-8 | Sample-9 |
|---|---|---|
| 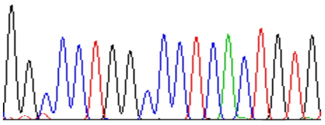 | 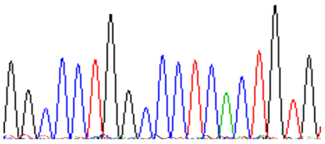 | 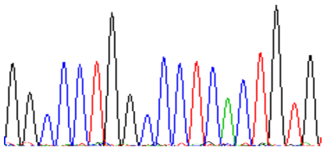 |
| Sample-10 | Sample-11 | Sample-12 |
| 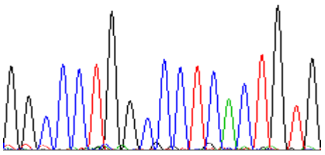 | 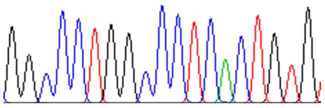 | 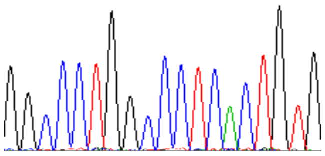 |
| Sample-13 | Sample-14 | Sample-15 |
| 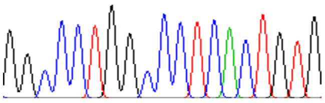 | 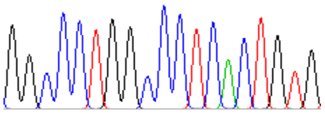 | 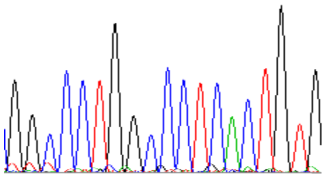 |
| Sample-16 | Sample-17 | Sample-18 |
| 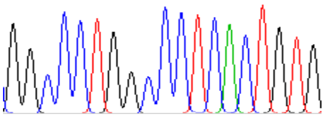 | 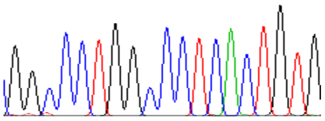 | 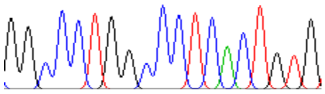 |

| Sample-19 | Sample-20 | Sample-21 |
|---|---|---|
| 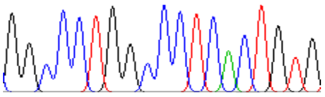 | 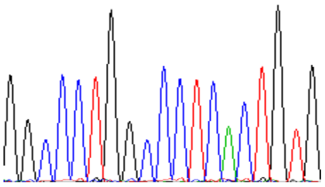 | 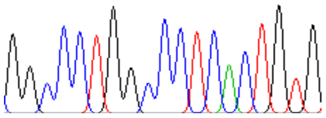 |
| Sample-22 | Sample-23 | Sample-24 |
| 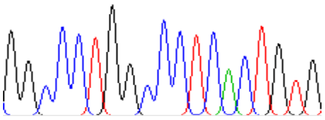 | 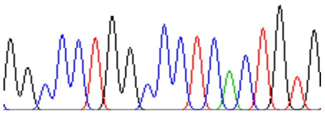 | 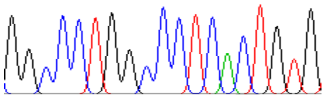 |
| Sample-25 | Sample-26 | Sample-27 |
| 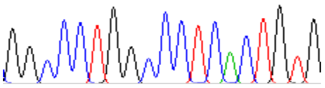 | 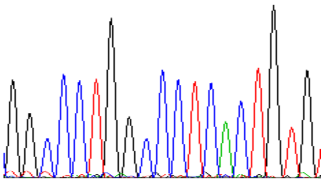 | 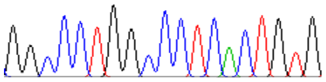 |
| Sample-28 | Sample-29 | Sample-30 |
| 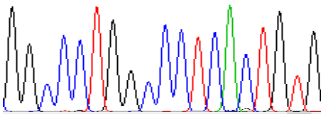 | 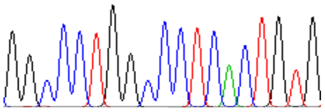 | 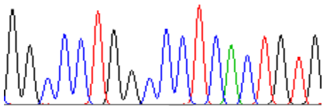 |

| Sample-31 | Sample-32 | Sample-33 |
| --- | --- | --- |
| 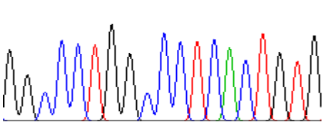 | 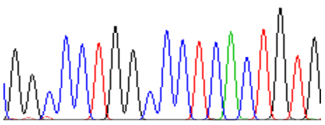 | 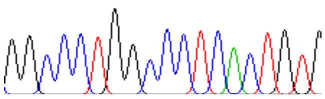 |
| Sample-34 | Sample-35 | Sample-36 |
| 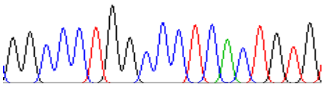 | 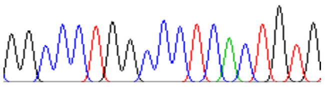 | 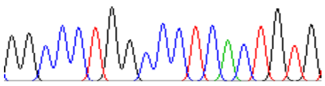 |
| Sample-37 | Sample-38 | Sample-39 |
| 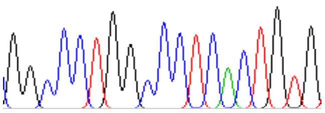 | 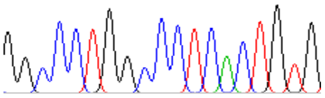 | 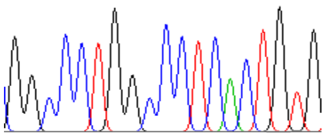 |
| Sample-40 | Sample-41 | Sample-42 |
| 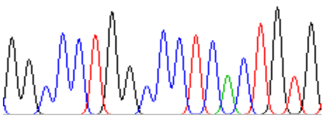 | 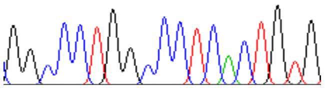 | 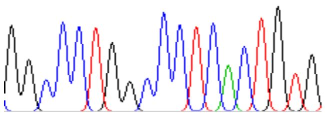 |

| Sample-43 | Sample-44 | Sample-45 |
|---|---|---|
| 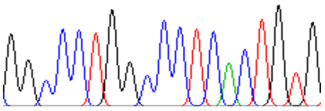 | 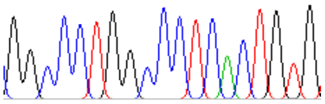 | 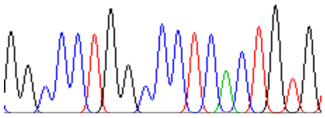 |
| Sample-46 | Sample-47 | Sample-48 |
| 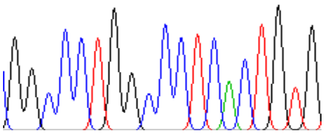 | 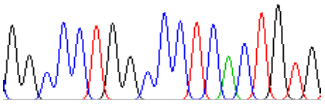 | 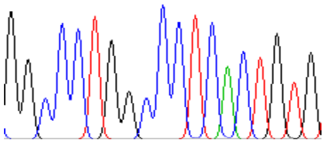 |
| Sample-49 | Sample-50 | Sample-51 |
| 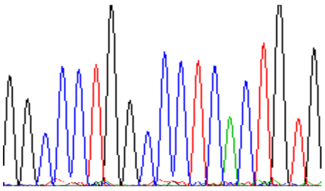 | 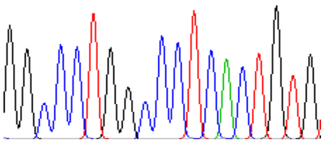 | 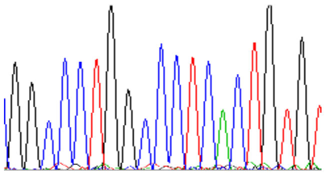 |
| Sample-52 | Sample-53 | Sample-54 |
| 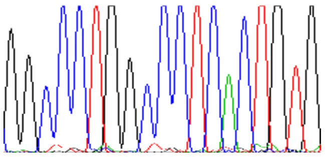 | 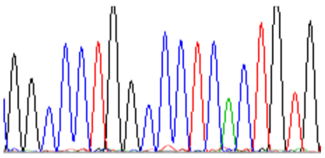 | 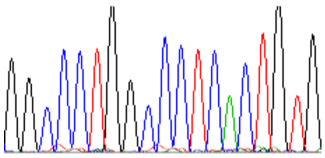 |

| Sample-55 | Sample-56 | Sample-57 |
|---|---|---|
| 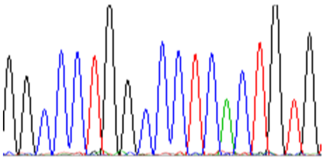 | 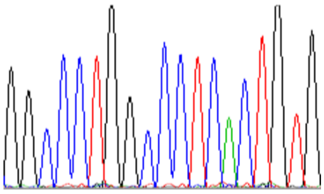 | 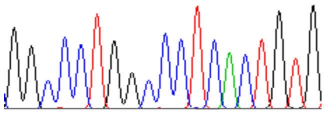 |
| Sample-58 | Sample-59 | Sample-60 |
| 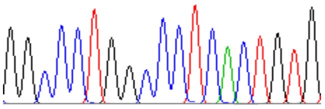 | 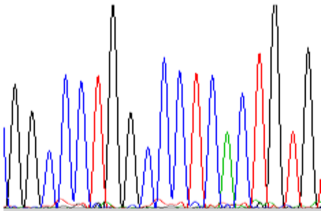 | 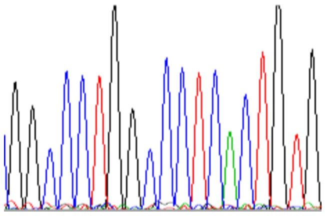 |
| Sample-61 | Sample-62 | Sample-63 |
| 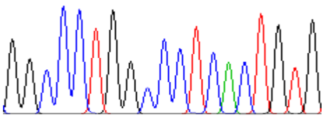 | 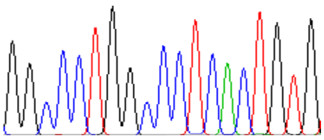 | 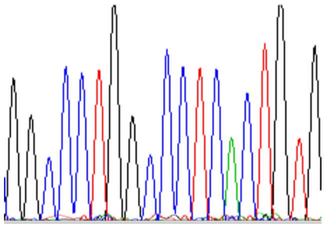 |
| Sample-64 | Sample-65 | Sample-66 |
| 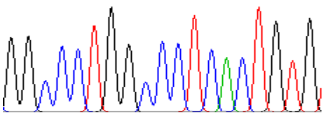 | 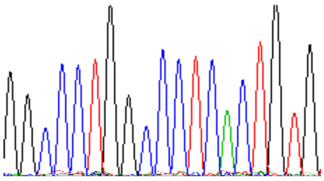 | 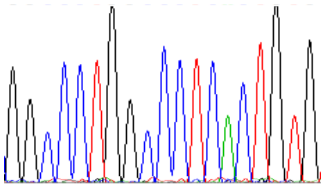 |

| Sample-67 | Sample-68 | Sample-69 |
| --- | --- | --- |
| 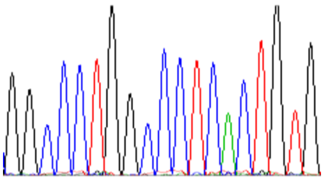 | 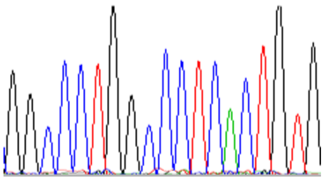 | 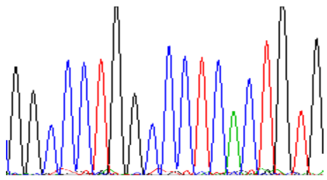 |
| Sample-70 | Sample-71 | Sample-72 |
| 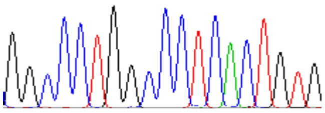 | 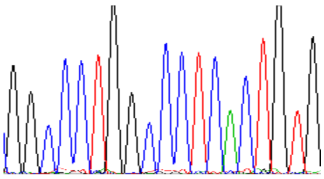 | 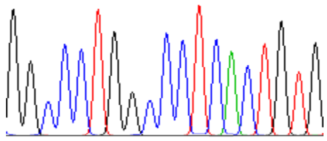 |
| Sample-73 | Sample-74 | Sample-75 |
| 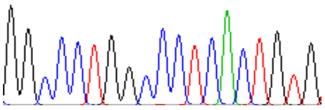 | 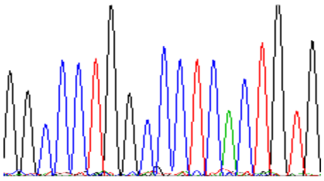 | 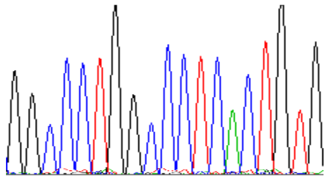 |
| Sample-76 | Sample-77 | Sample-78 |
| 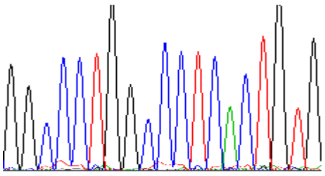 | 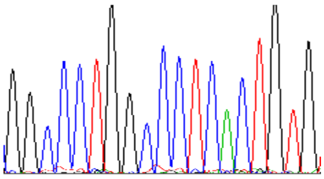 | 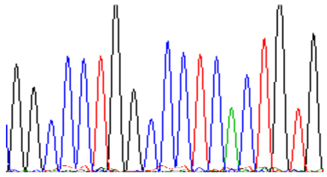 |

| Sample-79 | Sample-80 | Sample-81 |
|---|---|---|
| 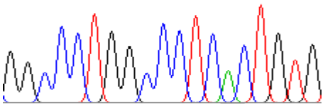 | 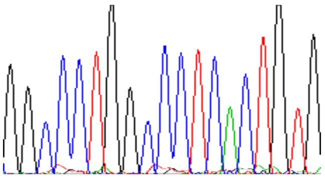 | 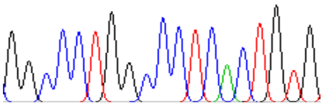 |
| Sample-82 | Sample-83 | Sample-84 |
| 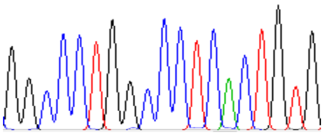 | 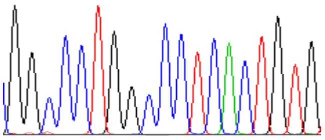 | 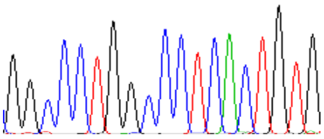 |
| Sample-85 | Sample-86 | Sample-87 |
| 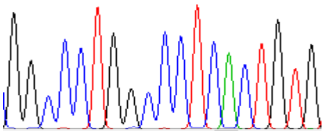 | 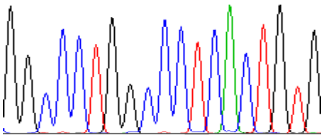 | 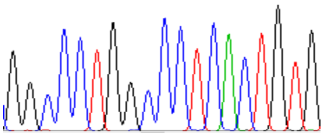 |
| Sample-88 | Sample-89 | Sample-90 |
| 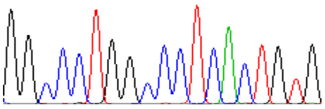 | 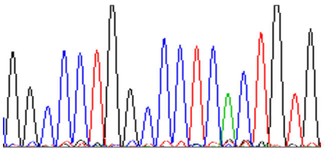 | 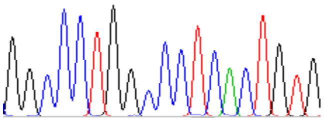 |

| Sample-91 | Sample-92 | Sample-93 |
| --- | --- | --- |
| 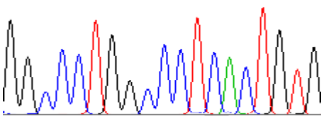 | 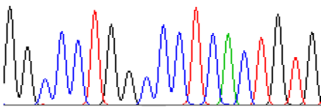 | 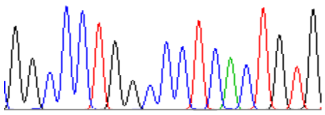 |
| Sample-94 | Sample-95 | Sample-96 |
| 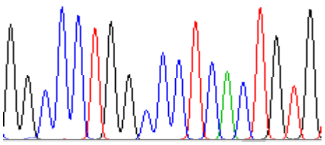 | 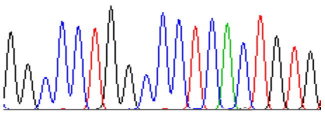 | 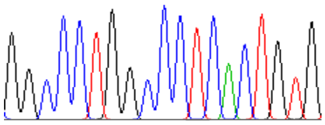 |
| Sample-97 | Sample-98 | Sample-99 |
| 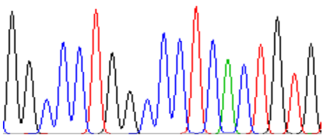 | 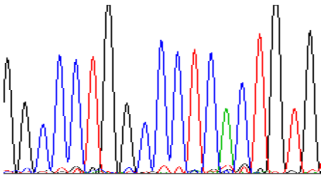 | 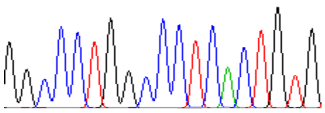 |
| Sample-100 | Sample-101 | Sample-102 |
| 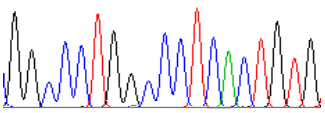 | 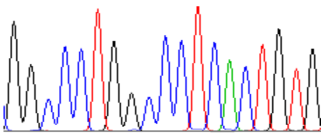 | 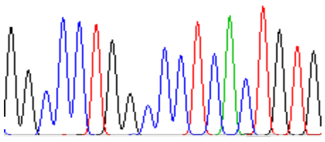 |
