# Supplementary material for: Rare variants in NRSN2 cause non-syndromic orofacial cleft through dysregulation of TGF-β signaling
Source: Genes Dis. 2025 Sep 23;13(3):101865. doi: 10.1016/j.gendis.2025.101865 (PMC12854861; doi:10.1016/j.gendis.2025.101865)
Supplement: Multimedia component 2 [file mmc2.docx]

**Supplementary data**

**Rare variants in *NRSN2* cause non-syndromic orofacial cleft through dysregulation of TGF-β signaling**

Xiaowen Zheng^a, b, c, 1^, Xuqin Liang^d, 1^, Xiantao Wu^d, 1^, Qing He^d, 1^, Chunyan Yin^c^, Yuhua Jiao^b, e^, Yanhao Wang^b, e^, Yuxia Hou^b, e, *^, Yi Ding^c, d, *^, Huaxiang Zhao^b, e, *^

1 These authors contributed equally to this work.

* Correspondence to:

[houyuxia@mail.xjtu.edu.cn](mailto:houyuxia@mail.xjtu.edu.cn) (Yuxia Hou), [dingyi1510@xjtu.edu.cn](mailto:dingyi1510@xjtu.edu.cn) (Yi Ding), and [huaxiangzhao@xjtu.edu.cn](mailto:huaxiangzhao@xjtu.edu.cn) (Huaxiang Zhao)

**The Supplementary data includes:**

- **Fig. S1.** Screening process to identify candidate rare variants in the multiplex family with NSOFC (refer to Fig. 1A).
- **Fig. S2.** Sanger sequencing chromatograms of the NSOFC patients in the multiplex family and sporadic cases (refer to Fig. 1A and B).
- **Fig. S3.** AlphaFold-3-predicted complex structures of NRSN2-TβRI and NRSN2-TβRII.
- **Fig. S4.** *NRSN2* variants exhibited reduced ability to repress Col2a1a expression in zebrafish embryos.
- **Fig. S5.** Schematic diagram illustrating the regulation of TGF-β signaling by NRSN2 and the potential pathogenic mechanism of *NRSN2* variants in OFC.
- **Table S1.** Information of the remaining 16 candidate genes/variants (refer to Fig. S1).
- **Table S2.** Genetic and clinical characteristics of NSOFC patients with *NRSN2* variants.
- **Table S3.** ACMG classification of p.W57fs, p.R104Q, and p.F183L variant in *NRSN2*.
- **Table S4.** Primers for PCR-Sanger sequencing of p.W57fs, p.R104Q and p.F183L variant in human *NRSN2* gene.
- **Supplemental Methods.**
- **Supplemental Material 1.** Sanger sequencing of *NRSN2* p.W57fs variant in 102 unaffected Han Chinese people (presented in a separate PDF file).


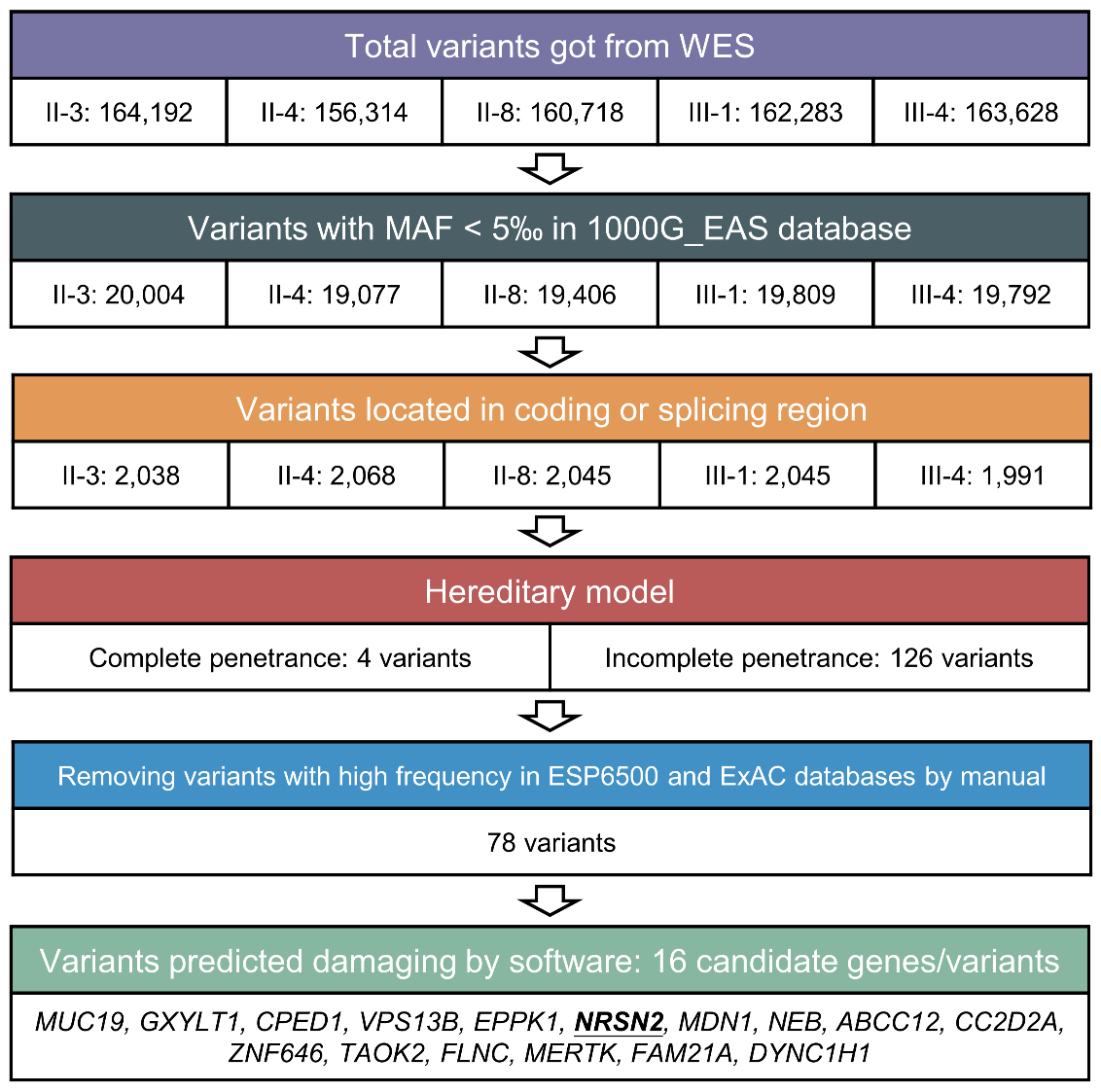


**Fig. S1. Screening process to identify candidate rare variants in the multiplex family with NSOFC (refer to Fig. 1A).**

Whole-exome sequencing (WES) was performed on both patients and unaffected members in Family 1. Variants with a low frequency (MAF < 5‰) in the 1000G_EAS database were selected for further analysis. Specifically, variants causing amino acid changes (missense, nonsense, insertion, deletion, *etc.*) or splicing variants were included for the subsequent analysis. Next, we employed two hereditary models, complete penetrance and incomplete penetrance, to narrow down the list of candidate variants, yielding 4 and 126 variants, respectively. To further refine the candidate variants, we manually cross-referenced these variants with other databases to eliminate those with high frequencies and got 78 remaining variants. Finally, we evaluated the potential effects of these variants by software, and identified 16 candidate genes/variants (refer to Table S1).


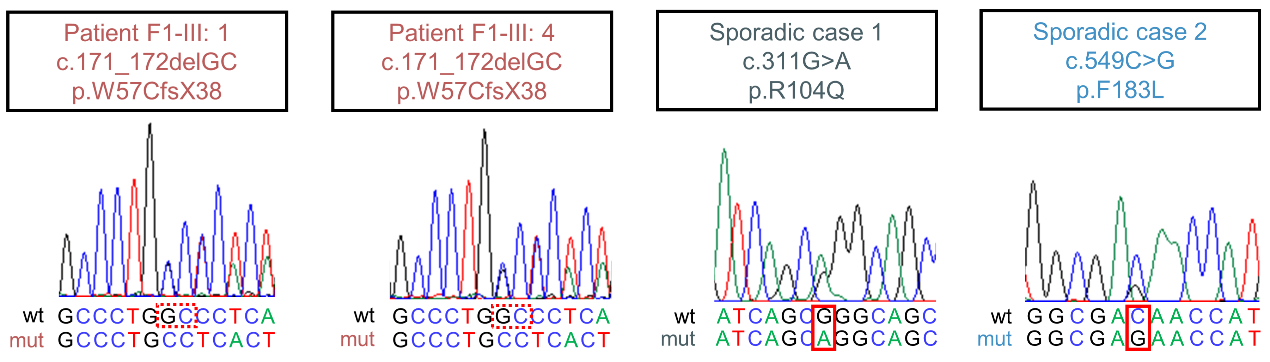


**Fig. S2. Sanger sequencing chromatograms of the NSOFC patients in the multiplex family and sporadic cases (refer to Fig. 1A and B).**

Dashed boxes in sequencing chromatograms denote deleted nucleotides, while solid boxes denote substituted nucleotides. wt, wild-type allele; mut, mutant allele.


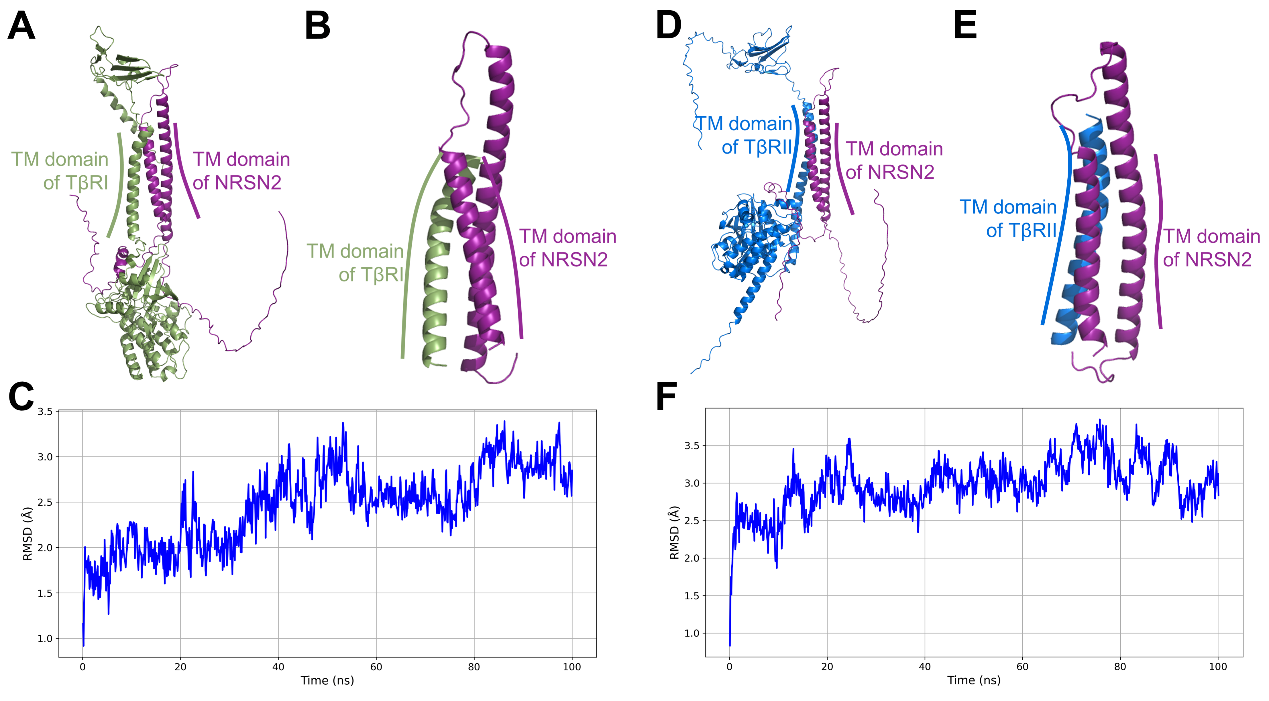


**Fig. S3. AlphaFold-3-predicted complex structures of NRSN2-TβRI and NRSN2-TβRII.**

**(A and B)** AlphaFold-3-predicted complex structures of NRSN2-TβRI. **A**, Full-length NRSN2 and TβRI; **B**, TM domains of NRSN2 (56-151 aa) and TβRI (123-156 aa). **(C)** Molecular dynamics simulation model of the TM domains of the NRSN2-TβRI complex. **(D and E)** AlphaFold-3-predicted complex structures of NRSN2-TβRII. **D**, Full-length NRSN2 and TβRII; **E**, TM domains of NRSN2 (56-151 aa) and TβRII (162-195 aa). **(F)** Molecular dynamics simulation model of the TM domains of the NRSN2-TβRII complex. TM, transmembrane region.


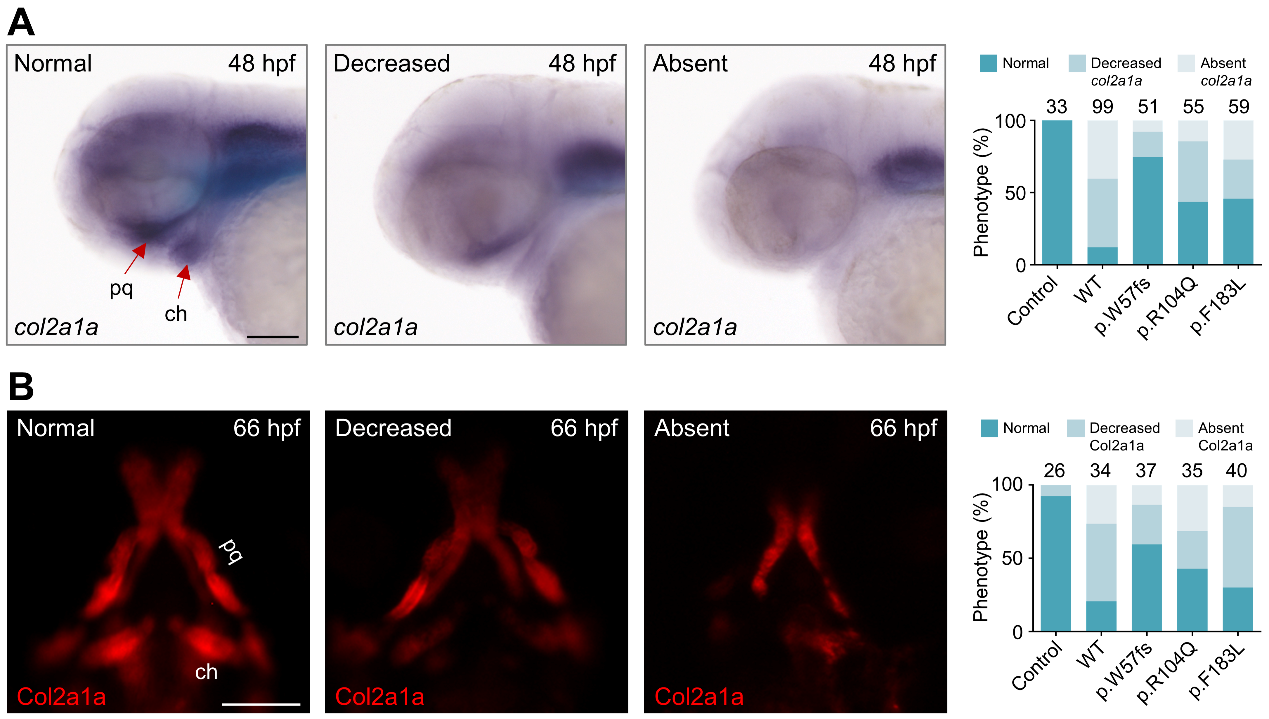


**Fig. S4. *NRSN2* variants exhibited reduced ability to repress Col2a1a expression in zebrafish embryos.**

Zebrafish embryos were microinjected as described in Fig. 1M and analyzed by **(A)** whole-mount *in situ* hybridization for *col2a1a* at 48 hpf and **(B)** immunofluorescence for Col2a1a protein at 66 hpf. Col2a1a was normally expressed in the palatoquadrate (pq) and ceratohyal (ch) cartilages and was suppressed by wild-type *NRSN2*. In contrast, all three *NRSN2* variants showed a reduced ability to repress Col2a1a expression. The number of embryos per condition is indicated on the top of each column. Scale bar, 100 μm.


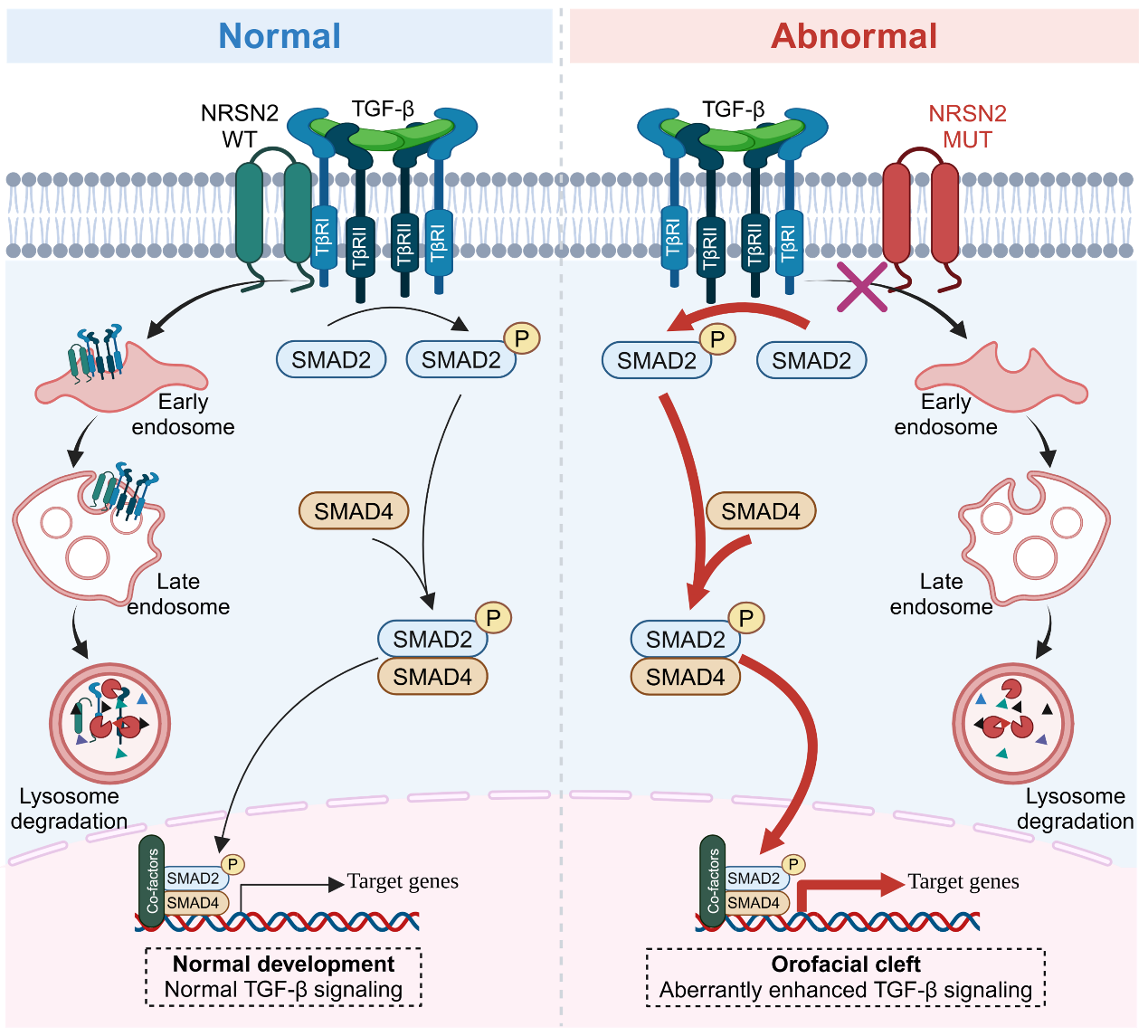


**Fig. S5. Schematic diagram illustrating the regulation of TGF-β signaling by NRSN2 and the potential pathogenic mechanism of *NRSN2* variants in OFC.**

**Left part:** Under normal physiological conditions, NRSN2 WT interacts with TGF-β receptors, TβRI and TβRII, and then facilitate their degradation *via* the endosome-lysosome pathway, which maintains the appropriate intensity of TGF-β signaling. **Right part:** NRSN2 MUT identified in this work exhibits impaired function compared to NRSN2 WT, leading to aberrantly enhanced TGF-β signaling, which contributes to the formation of OFC. Bold red arrows indicate aberrantly enhanced signaling transduction. NRSN2 WT, wild-type NRSN2; NRSN2 MUT, NRSN2 mutant; P, phosphorylation; OFC, orofacial cleft. Schematic illustrations were created with BioRender.com.

**Table S1. Information of the remaining 16 candidate genes/variants (refer to Fig. S1)**

| **Gene** | **Type** | **Het/Hom** | **Position change**  **in chromosome** | **cDNA change** | **Amino acid**  **substitution** | **Information in databases** | | | **Predicted pathogenicity** | |
| --- | --- | --- | --- | --- | --- | --- | --- | --- | --- | --- |
|  |  |  |  |  |  | **dbSNP** | **1000G**  **(All)** | **1000G**  **(Asian)** | **SIFT** | **PolyPhen-2** |
| *NRSN2* | Frameshift | Het | chr20:330457GGC>G | c.171_172delGC | p.Trp57fs | NR | NR | NR | / | / |
| *EPPK1* | Frameshift | Het | chr8:144947106GC>G | c.315delG | p.Leu106fs | rs781831326 | NR | NR | / | / |
| *CPED1* | Frameshift | Het | chr7:120773900AC>A | c.1602delC | p.Asn534fs | rs762152754 | NR | NR | / | / |
| *GXYLT1* | Nonsense | Het | chr12:42538352C>A | c.97G>T | p.Gly33* | rs1262821887 | NR | NR | / | / |
| *MUC19* | Stop-lost | Het | chr12:40882632A>G | c.15878A>G | p.Ter5293Trpext*? | rs200982622 | NR | NR | / | / |
| *MDN1* | missense | Het | chr6:90397121G>A | c.11392C>T | p.Arg3798Trp | rs148868949 | 0.0006 | 0 | 0.007(D) | 0.992(D) |
| *NEB* | missense | Het | chr2:152512419C>T | c.6614G>A | p.Arg2205His | rs752349680 | NR | NR | 0.005,0.004,0.004,0.005(D,D,D,D) | 0.87(P) |
| *ABCC12* | missense | Het | chr16:48177911G>A | c.185C>T | p.Pro62Leu | rs140339167 | 0.0006 | 0 | 0.001(D) | 0.991(D) |
| *CC2D2A* | missense | Het | chr4:15538580G>A | c.1645G>A | p.Glu549Lys | rs368788560 | NR | NR | 0.013(D) | 0.897,0.957(P,D) |
| *ZNF646* | missense | Het | chr16:31091801C>G | c.4156C>G | p.His1386Asp | rs771172580 | NR | NR | 0.04(D) | 0.951(D) |
| *TAOK2* | missense | Het | chr16:30002434C>G | c.2695C>G | p.Gln899Glu | rs763077540 | NR | NR | 0.005(D) | 0.99(D) |
| *FLNC* | missense | Het | chr7:128481550G>C | c.2050G>C | p.Val684Leu | rs769221710 | NR | NR | 0.052,0.048(T,D) | 0.535,0.203(P,B) |
| *MERTK* | missense | Het | chr2:112786223C>T | c.2782C>T | p.Arg928Trp | rs750606891 | NR | NR | 0.0(D) | 0.995(D) |
| *FAM21A* | missense | Het | chr10:51827941A>G | c.80A>G | p.Glu27Gly | NR | NR | NR | 0.001,0.001,0.001(D,D,D) | 0.897,0.474,0.928(P,P,D) |
| *DYNC1H1* | missense | Het | chr14:102494086G>A | c.9179G>A | p.Arg3060His | rs1408182872 | NR | NR | 0.006(D) | 0.885(P) |
| *VPS13B* | Splicing | Com-Het | chr8:100844569 A>ATT,ATTT | c.9406-4_9406-3dupTT | / | NR | NR | NR | / | / |

The version of chromosome database used in this study is GRCh37/hg19.

“NR”: Not reported; “/”: Cannot be predicted.

“Het”: Heterozygous; “Hom”: Homozygous; “Com-Het”: Compound heterozygous.

“D”: Damaging; “T”: Tolerated; “P”: Possibly damaging; “B”: Benign.

**Table S2. Genetic and clinical characteristics of NSOFC patients with *NRSN2* variants**

|  | Patient F1-III: 1  p.W57fs | Patient F1-III: 4  p.W57fs | Sporadic case 1  p.R104Q | Sporadic case 2  p.F183L |
| --- | --- | --- | --- | --- |
| **Variant annotation** | | | | |
| Het/Hom | Het | Het | Het | Het |
| Chromosome | 20 | 20 | 20 | 20 |
| Position (GRCh37) | 330458 | 330458 | 333975 | 334213 |
| nt ref | GC | GC | G | C |
| nt alt | del | del | A | G |
| Protein ref | p.W57 | p.W57 | p.R104 | p.F183 |
| Protein alt | p.W57CfsX38 | p.W57CfsX38 | p.Q104 | p.L183 |
| dbSNP | NR | NR | rs199769761 | rs764415866 |
| gnomAD-Exomes (Asian) | NR | NR | 0.00012 | 0.00018 |
| ALFL (East Asian) | NR | NR | 0.000 | 0.00 |
| TOPMed (Global) | NR | NR | 0.000008 | 0.000053 |
| **Clinical presentation** | | | | |
| Gender | Male | Female | Female | Male |
| Age of recruitment | 8 years old | 12 years old | 24 years old | One year old |
| Cleft lip | A right-side cleft lip | A left-side cleft lip | A left-side cleft lip | A right-side cleft lip |
| Cleft palate | - | - | - | - |
| Congenital heart disease | - | - | - | - |
| Intelligence | Normal | Normal | Normal | Unable to assess |
| Motor development status | Normal | Normal | Normal | Normal |
| Other congenital defect | - | - | - | - |

“Het”: Heterozygous; “Hom”: Homozygous; “nt”: Nucleotide; “ref”: Reference sequence; “alt”: Alternate sequence; “NR”: Not reported; “-”: Cannot be detected.

**Table S3. ACMG classification of p.W57fs, p.R104Q, and p.F183L variant in *NRSN2***

| **Variants** | **ACMG classification** | **Evidence of pathogenicity** |
| --- | --- | --- |
| p.W57fs | Pathogenic | PVS1 + PS3 +PM2 + PP3 |
| p.R104Q | Pathogenic | PS3 + PS4 + PP3 |
| p.F183L | Pathogenic | PS3 + PS4 + PP3 |

Note:

- PVS1: The p.W57fs variant in *NRSN2* is a frameshift variant.
- PS3: Functional studies *in vitro* and *in vivo* supporting the damaging effect on the gene.
- PS4: The prevalence of the variant in affected individuals is increased compared with controls. The prevalence of p.R104Q and p.F183L are derived from the gnomAD-Exomes database (Asian), and the OR for p.R104Q and p.F183L are 59.61 (7.13 ~ 498.39) and 39.73 (5.00 ~ 315.75), respectively.
- PM2: Absent from controls in various databases.
- PP3: Multiple lines of computational evidence support a deleterious effect.

**Table S4. Primers for PCR-Sanger sequencing of p.W57fs, p.R104Q and p.F183L variant in human *NRSN2* gene**

| **Primer name** | **Primer sequence (5’-3’)** | **PCR product size** | **Purpose** |
| --- | --- | --- | --- |
| NRSN2-0451-F | GCTATGCGTGTAGGGTG | 462 bp | PCR-Sanger sequencing of p.W57fs variant |
| NRSN2-0451-R | GGGTTTTCTGTTGCTTG |  |  |
| NRSN2-3975-F | TGTGGCGGCTCTGA | 220 bp | PCR-Sanger sequencing of p.R104Q variant |
| NRSN2-3975-R | CTCTGCCTTGGTGTCC |  |  |
| NRSN2-4213-F | CAGCAGTTGTCACCCAT | 143 bp | PCR-Sanger sequencing of p.F183L variant |
| NRSN2-4213-R | GGACCCACATCTTAGGC |  |  |

**Supplemental methods**

**Clinical samples**

Given that NSOFC is a complex congenital defect influenced by both genetic and environmental factors, multiplex families are believed to have a stronger genetic contribution compared to sporadic cases ^1^. To investigate potential genetic causes of NSOFC, we have recruited 10 multiplex families with NSOFC over the past four years, each with at least two affected members. In addition, 138 sporadic NSOFC patients were included. A control group of 102 unaffected individuals was also recruited. All human samples of this work were obtained from individuals of Han population in China. Two surgeons thoroughly examined all patients and controls to ensure accurate identification of craniofacial and other organic phenotypes. Genomic DNA was extracted from peripheral blood using the QIAamp DNA Blood Mini Kit (Qiagen, #51006).

**Whole-exome sequencing, target-region sequencing, and Sanger sequencing**

Whole-exome sequencing (WES) of the 10 multiplex families with NSOFC was performed using the BGISEQ-500 platform (BGI *Inc.*, China), and the genomic variations were mapped to the human GRCh37/hg19 reference genome. Our strategy for screening candidate genes/variants in each family followed our previous work ^2^. First, we excluded variants with an allele frequency ≥ 0.5‰, as such high-frequency variants are unlikely to be pathogenic. Next, we focused on variants located in coding or splicing regions, as these are more likely to alter protein function. Third, we applied two inheritance models—complete and incomplete penetrance—to further prioritize candidate variants, since NSOFC can exhibit incomplete penetrance ^3;4^. Fourth, we manually reviewed the remaining variants across multiple databases to minimize the risk of missing relevant information. Finally, we used bioinformatic tools to predict the functional impact of each variant, retaining only those predicted to be damaging.

Once the candidate gene was identified in the multiplex families, we conducted target-region sequencing (TRS) of the exons of the candidate gene to validate its presence in 138 sporadic NSOFC patients. All variants identified through WES and TRS were confirmed using PCR-Sanger sequencing with specific primers (primers for three *NRSN2* variants are listed in **Table S4**). If the candidate variant was not reported in any genomic databases, we additionally performed PCR-Sanger sequencing in the control group of 102 healthy individuals, to confirm the rarity of the variant.

**Bioinformatic analysis**

We utilized SIFT and PolyPhen-2 to assess the potential pathogenicity of the candidate variants as previously described ^5^. AlphaFold-3 ^6^ was employed to model the complex structure between NRSN2 and other transmembrane receptor proteins, and CHARMM-GUI Membrane Builder ^7^ was used to simulate molecular dynamics to evaluate the stability of the complex structure.

**Cloning**

The full-length wild-type *NRSN2* (NM_001323680) was cloned into pCS2 vectors (containing a carboxyl-terminal Flag or myc tag) and pmCherry-N1 vectors (containing a carboxyl-terminal Cherry). We employed site-directed mutagenesis to generate the p.W57fs, p.R104Q, and p.F183L mutants of NRSN2. Dr. Ye-guang Chen from Tsinghua University kindly provided us with TβRI, TβRII, RAB5A, RAB7A, RAB11A, and LAMP1 constructs. The sequences of all constructs used in this work were confirmed through directed Sanger-sequencing.

**Cell culture and transfection**

HEK-293T and HeLa cells were cultured in DMEM medium supplemented with 10% fetal bovine serum (BI, #04-001-1ACS) and 1% penicillin-streptomycin, in a 5% CO_2_ incubator at 37°C. Both cells were transfected using Lipo3000 reagent (Thermo, #L3000015), following the manufacturer’s instructions.

**Immunoprecipitation and immunoblotting**

The procedures for immunoprecipitation (IP) and immunoblotting (IB) were performed as described previously ^8^. In brief, cells were lysed at 4°C for 10 min with the lysis buffer and aliquots of total cell lysates containing equal amounts of total proteins were subjected to IP or IB. For IP, the cell lysates were precleared with protein A/G PLUS-Agarose beads (Santa Cruz, #sc-2003) for 4 hours at 4°C, followed by incubation with the anti-FLAG or anti-MYC antibodies (Santa Cruz, #sc-166355; Santa Cruz, #sc-40) and protein A-Sepharose beads at 4°C overnight. The immunocomplexes were isolated by centrifugation, washed with the lysis buffer, and subjected to SDS-PAGE and IB. The antibodies used for IB were: HA rabbit Ab (CST, #3724), FLAG mouse Ab (Origene, #TA180144), MYC mouse Ab (CST, #2276), HIS mouse Ab (Santa Cruz, #sc-8036), pSMAD2 rabbit Ab (CST, #3108), SMAD2 rabbit Ab (CST, #3102), β-ACTIN mouse Ab (Origene, #TA811000), and TUBULIN mouse Ab (Origene, #BM753S).

**Immunofluorescence**

HeLa cells transfected with the indicated constructs were harvested for immunofluorescence (IF) analysis. The subcellular localization of the proteins was visualized using direct GFP, Cherry, and BFP signals with an Olympus FV3000 confocal laser scanning microscope as described in our previous study ^9^.

For IF analysis of whole-mount zebrafish embryos, embryos were harvested at 66 hpf, fixed overnight at 4 °C in 4% paraformaldehyde (PFA), dehydrated with methanol, and stored at -20 °C for at least 2 h. Following rehydration, embryos were permeabilized with proteinase K (10 μg/mL) for 30 min. After blocking (2% BSA, 5% goat serum, and 0.1% Tween-20 in PBS) for 1 h, embryos were incubated with anti-COL2A1 antibody (DSHB, #II-II6B3) as previously described ^10^. Images were acquired using a ZEISS Axio Zoom V16 microscope.

**Zebrafish**

The wild-type embryos used in this study were Tübingen (TU) strains and raised in Holtfreter’s solution at 28.5°C. All zebrafish embryos were staged according to morphological characteristics as described ^11^.

**mRNA synthesis, microinjection, Alcian blue staining, and whole-mount *in situ* hybridization**

Wild-type or p.W57fs or p.R104Q or p.F183L *NRSN2* in pCS2 vector was linearized and transcribed using the mMESSAGE mMACHINE^TM^ SP6 Transcription Kit (Invitrogen, #AM1340). A total of 400 pg mRNA was microinjected into zebrafish embryos at one-cell stage and the embryos were cultured till 5 days post-fertilization (dpf). The zebrafish embryos were subsequently fixed overnight in 4% PFA and stained using a 0.1 mg/ml Alcian blue reagent (Sigma, #5268). After destaining, the embryos were further fixed using glycerin and prepared for imaging. For whole-mount *in situ* hybridization, embryos were collected at 48 hpf and processed using a standard protocol for *col2a1a*, as described in our previous studies ^12^. The images were acquired using a ZEISS Axio Zoom V16 microscope, following the protocol described in our previous study ^9^.

**References for Supplementary data**

1. Diaz Perez KK, Curtis SW, Sanchis-Juan A, et al. Rare variants found in clinical gene panels illuminate the genetic and allelic architecture of orofacial clefting. *Genet Med.* 2023:100918.

2. Zhong W, Zhao H, Huang W, et al. Identification of rare PTCH1 nonsense variant causing orofacial cleft in a Chinese family and an up-to-date genotype-phenotype analysis. *Genes Dis.* 2021;8(5):689-697.

3. Basha M, Demeer B, Revencu N, et al. Whole exome sequencing identifies mutations in 10% of patients with familial non-syndromic cleft lip and/or palate in genes mutated in well-known syndromes. *J Med Genet.* 2018;55(7):449-458.

4. Cox LL, Cox TC, Moreno Uribe LM, et al. Mutations in the Epithelial Cadherin-p120-Catenin Complex Cause Mendelian Non-Syndromic Cleft Lip with or without Cleft Palate. *Am J Hum Genet.* 2018;102(6):1143-1157.

5. Zhao H, He Q, Wu X, et al. Identification of rare loss-of-function variants in FAM3B associated with non-syndromic orofacial clefts. *Genomics.* 2023;115(3):110630.

6. Abramson J, Adler J, Dunger J, et al. Accurate structure prediction of biomolecular interactions with AlphaFold 3. *Nature.* 2024;630(8016):493-500.

7. Wu EL, Cheng X, Jo S, et al. CHARMM-GUI Membrane Builder toward realistic biological membrane simulations. *J Comput Chem.* 2014;35(27):1997-2004.

8. Zhang F, Zhu X, Wang P, et al. The cytokine FAM3B/PANDER is an FGFR ligand that promotes posterior development in Xenopus. *Proc Natl Acad Sci U S A.* 2021;118(20):e2100342118.

9. He Q, Hao X, Bao S, et al. A392V and R945X mutations cause orofacial clefts via impairing PTCH1 function. *Genomics.* 2022;114(6):110507.

10. Ning G, Liu X, Dai M, Meng A, Wang Q. MicroRNA-92a upholds Bmp signaling by targeting noggin3 during pharyngeal cartilage formation. *Dev Cell.* 2013;24(3):283-295.

11. Kimmel CB, Ballard WW, Kimmel SR, Ullmann B, Schilling TF. Stages of embryonic development of the zebrafish. *Dev Dyn.* 1995;203(3):253-310.

12. He Q, Yu M, Jiao Y, et al. Exome Sequencing Reveals the Genetic Architecture of Non-syndromic Orofacial Clefts and Identifies BOC as a Novel Causal Gene. *Adv Sci (Weinh).* 2025:e12073.
